# Beyond Advanced Cardiac Life Support: Dual-sequential Defibrillation for Refractory Ventricular Fibrillation after Witnessed Cardiac Arrest in the Emergency Department

**DOI:** 10.7759/cureus.3717

**Published:** 2018-12-11

**Authors:** Kenneth L Frye, Ademola Adewale, Elijah Kennedy, Lisa O'Grady

**Affiliations:** 1 Emergency Medicine, Florida Hospital, Orlando, USA; 2 Emergency Medicine, Florida Hospital, Orlando , USA

**Keywords:** dual sequential defibrillation, refractory ventricular fibrillation, emergency medicine, acls, resuscitation, cardiac arrest, point-of-care ultrasound, pocus

## Abstract

Refractory ventricular fibrillation is a rare condition seen in both in-hospital and out-of-hospital cardiac arrest. A 56-year-old male was identified to have refractory ventricular fibrillation after an in-hospital cardiac arrest with multiple unsuccessful standard defibrillation attempts that was converted with dual-sequential defibrillation (DSD) to normal sinus rhythm. Advanced cardiac life support (ACLS) is the most widely used algorithmic treatment approach for various cardiopulmonary emergencies but has yet to provide recommendations for the treatment of refractory ventricular fibrillation. DSD may be a viable treatment strategy for refractory ventricular fibrillation when ACLS recommendations are ineffective.

## Introduction

Cardiac arrest due to refractory ventricular fibrillation is a rare, life-threatening condition, seen in less than 10% of out-of-hospital cardiac arrests [1]. The term refractory ventricular fibrillation is used when there have been multiple failed attempts (two or more) at correcting ventricular fibrillation during cardiopulmonary resuscitation (CPR) [2]. Advanced cardiac life support (ACLS) guidelines help providers with clinical decisions while managing cardiac arrest patients but provide no recommendations for refractory ventricular fibrillation. Here we present a case detailing a potential treatment strategy for refractory ventricular fibrillation using dual-sequential defibrillation (DSD) once the ACLS algorithm pathways have been exhausted.

## Case presentation

**Figure 1:**
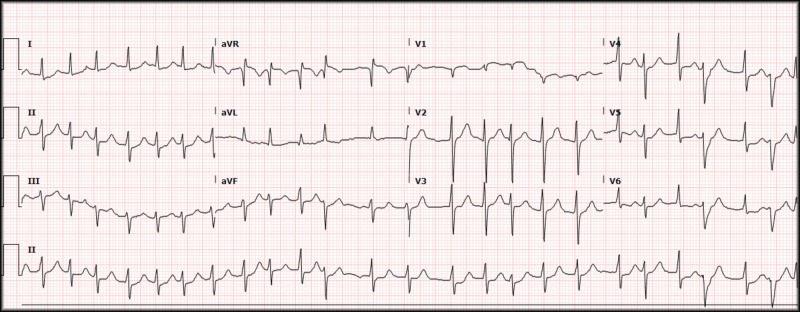
Electrocardiogram, atrial fibrillation. Rate 147, atrial fibrillation, left axis deviation, QRS 84 ms, QTc 404, ST-segments with minimal depression in lateral leads.

A 56-year-old male presented to the emergency department (ED) with significant substernal chest pain starting 30 minutes prior to arrival. The patient was immediately brought back to an exam room after an electrocardiogram (ECG) was performed and was seen by a provider within 10 minutes of registration (Figure 1).

**Figure 2:**
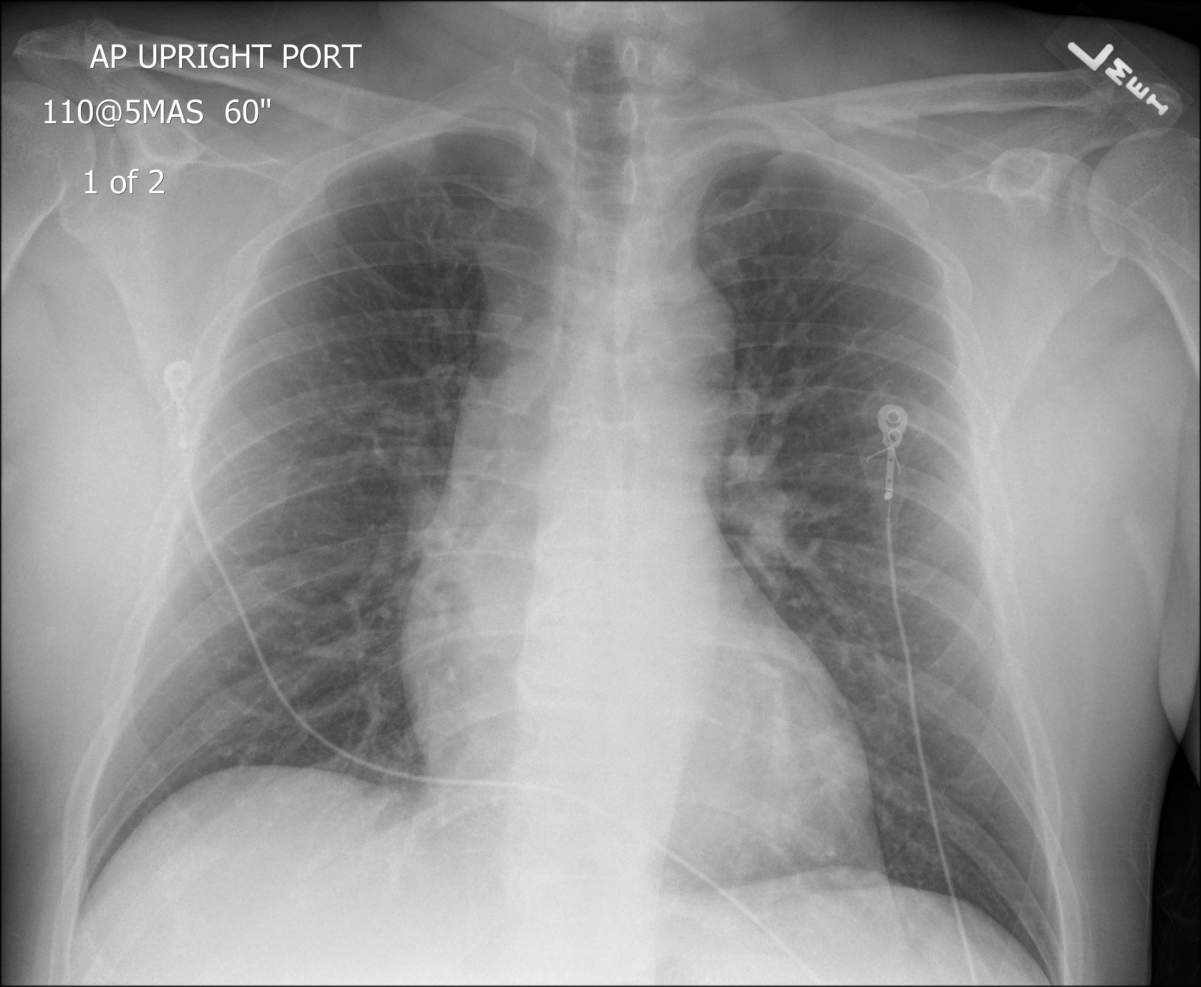
Chest X-ray. Portable chest X-ray, anterior-posterior orientation.

The initial ECG revealed atrial fibrillation with a rapid ventricular response, rate of 147, with minimal ST depression within the lateral leads but was without apparent ST-segment elevation. On initial assessment, the patient had point-of-care labs immediately drawn (a basic metabolic panel and troponin), and a chest X-ray performed to evaluate for a possible aortic dissection which revealed no gross abnormalities (Figure 2).

With a non-diagnostic chest X-ray alternative diagnoses were pursued. The ultrasound fellow in the department was consulted for an immediate cardiac ultrasound for evaluation of right heart strain secondary to pulmonary embolism. During the bedside cardiac ultrasound, the patient experienced ventricular fibrillation (Video 1), and CPR was immediately started.

**Video 1:** Ventricular fibrillation. Point-of-care ultrasound (POCUS), phased-array probe, subxiphoid view.

**Figure 3:**
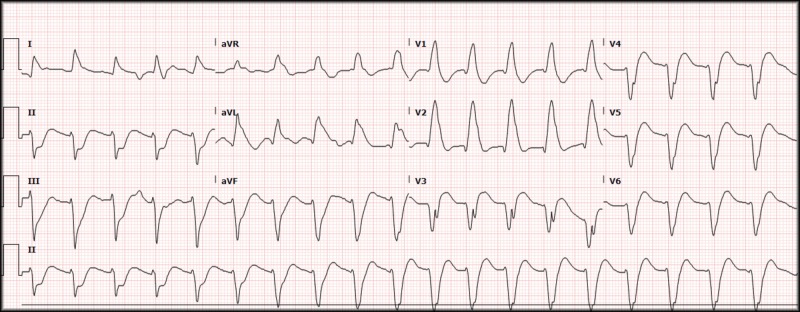
Electrocardiogram, wide complex tachycardia. Ventricular rate 116, QRS 200 ms.

The ACLS algorithm was followed for pulseless ventricular fibrillation, and the patient received multiple rounds of epinephrine, 450 mg of amiodarone (300 mg and then 150 mg), and three conventional defibrillations with increasing joules at 150 J, 200 J, and 200 J (the departmental defibrillators are biphasic and have a maximum output of 200 J). The patient continued with ventricular fibrillation throughout the ACLS algorithm, and the decision was made to attempt DSD. The patient had a second set of pads applied in the anterior-posterior orientation in addition to the conventional right upper chest and left lateral chest with successful conversion of the ventricular fibrillation. The patient was additionally given Lidocaine, 100 mg, due to a wide-complex tachycardia and apparent non-responsiveness to the previously given amiodarone (Figure 3). An improvement was noted after the use of Lidocaine, and a Lidocaine drip was started.

**Figure 4:**
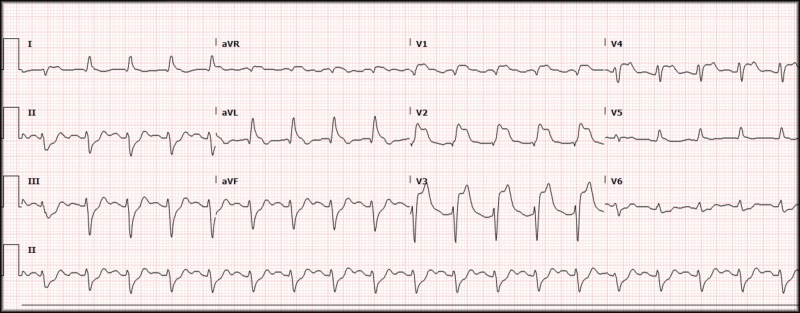
Electrocardiogram, STEMI. Rate 114, sinus tachycardia, left axis deviation, PR 156, QRS 116, QTc 391, ST-segment elevations in aVR, V1, V2, V3, and V4 with reciprocal depressions in II, III, and aVF. STEMI: ST-elevation myocardial infarction

Once the patient was stabilized, he was taken for computed tomography (CT) imaging to further evaluate for the possibility of a pulmonary embolism. In the CT room, he developed bradycardia and subsequently lost his pulse. CPR was again started, the patient was given atropine, and return of spontaneous circulation (ROSC) was achieved shortly after. The CT scan did not reveal any evidence of aortic dissection or pulmonary embolism and a repeat ECG was performed which showed a persistent wide complex tachycardia with no obvious ST-segment changes. Due to the morphology of the QRS complexes and length of resuscitation time from initial arrest (nearing 90 minutes), tissue plasminogen activator (tPA) was used as a thrombolytic for what was presumed to be a large vessel occlusion myocardial infarction. Hundred milligram of tPA was administered (50 mg as a bolus and 50 mg as a drip given over 60 minutes) with an apparent reperfusion rhythm followed by a "normal" appearing sinus tachycardia. Another ECG was repeated which revealed sinus tachycardia, at a rate of 114, now with ST-segment elevations present in aVR, V1, V2, V3, and V4 with depressions in leads II, III, and aVF (Figure 4).

Given the patient's persistent elevations despite thrombolytic therapy, interventional cardiology was consulted, and the patient was transferred to a tertiary care facility for cardiac catheterization revealing a thrombotic occlusion in the proximal left anterior descending coronary artery.

After transfer to the tertiary care facility for cardiac catheterization, the patient developed cardiogenic shock. The patient was started on ionotropic medications with no improvement, and he was placed on venous-arterial extracorporeal membrane oxygenation (ECMO) therapy (~20 hours after his cardiac arrest). Before initiation of ECMO, the patient was awake, alert, and following simple commands (although still intubated). Unfortunately, despite ECMO support, his cardiac function did not improve, and the patient was not a candidate for cardiac transplantation. Seventeen days after the patient's initial presentation to the ED, the family decided to withdraw care. The patient was extubated, had ECMO discontinued, and time of death was documented shortly after.

## Discussion

Cardiac arrest (in-hospital or out-of-hospital) is a frequent presentation in the emergency department with an algorithmic treatment approach (ACLS). In the event of algorithm failure, i.e., refractory ventricular fibrillation in the setting of multiple unsuccessful defibrillation attempts, new concepts, and treatment modalities should be considered. One such treatment modality that should be considered is DSD as there is evidence that suggests its use may be associated with improved rates of intact neurologic survival in patients with refractory ventricular fibrillation [3].

**Figure 5:**
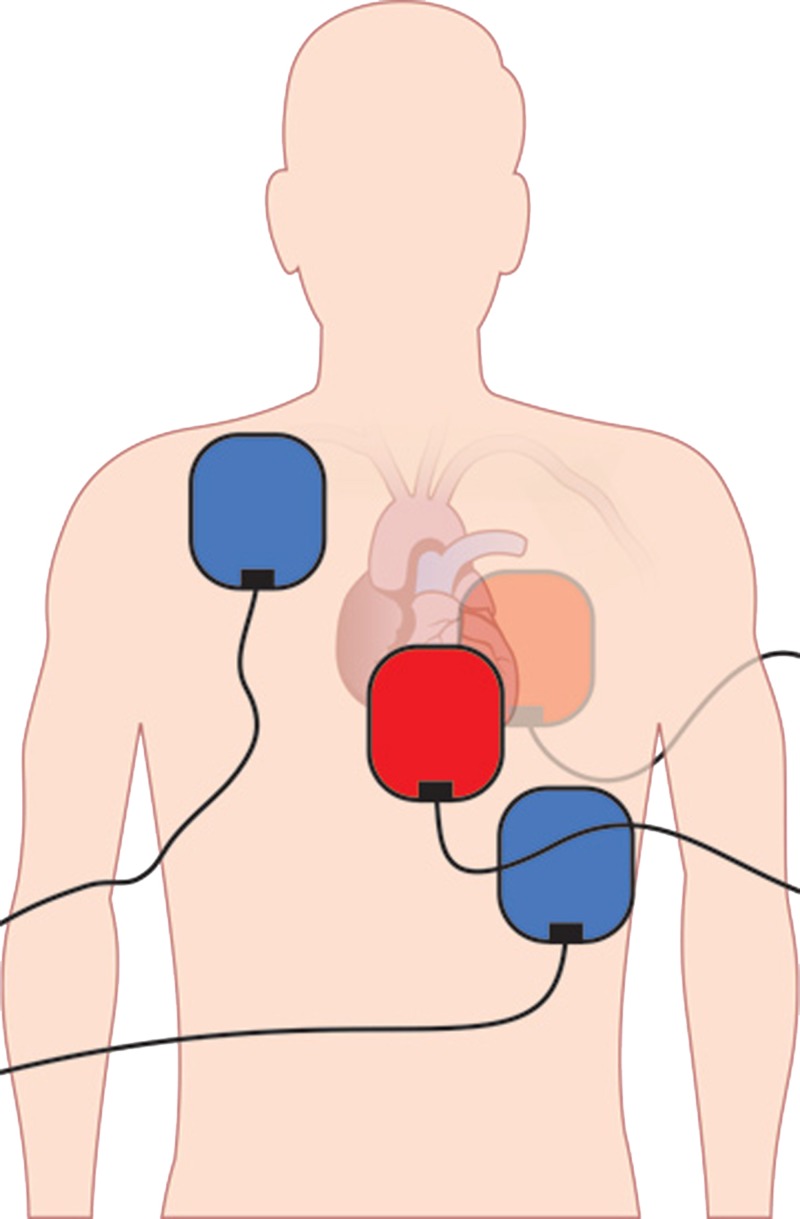
Diagram of appropriate pad placement. Blue Pads: conventional orientation, Red Pads: anterior-posterior orientation.

DSD describes the use of two separate defibrillator units with defibrillator pads placed in the conventional right upper chest-left lateral lower chest orientation with an additional set of pads placed in an anterior-posterior orientation (Figure 5) [4].

The defibrillator units are then activated by the same user paying particular attention to delivering the defibrillations as close to the same time as possible. Given the impossible nature of true "simultaneous" defibrillation without interconnected defibrillation units, the term dual-sequential defibrillation has been used to more accurately describe the firing of each unit in close succession. DSD has been postulated to be effective for the treatment of refractory ventricular fibrillation through multiple theoretical mechanisms including the following:

(1) Sequential shocks may lower the defibrillation threshold [5,6].

(2) Defibrillation may be a weight-based treatment, and therefore with larger patients, more joules are needed [7,8].

(3) By utilizing multiple defibrillation pads, the vector of electricity is changed, increasing the amount and duration of myocardium that undergoes defibrillation [9].

There have been multiple previously documented case reports with successful conversion of refractory ventricular fibrillation via the use of DSD. No specific recommendations have been established, but as more cases are documented, treatment patterns, processes, and protocols can be created. Despite the multiple case reports detailing the effectiveness of DSD, there have been no formal studies to date performed in the ED investigating its utility. There are numerous barriers to the development of formal studies including the rarity of refractory ventricular fibrillation, identification of refractory vs. recurrent ventricular fibrillation, as well as a scarcity of established protocols which utilize or suggest the use of DSD [1].

Identification of refractory ventricular fibrillation can be difficult in the setting of an active resuscitation. Previous studies have suggested refractory and recurrent ventricular fibrillation (ventricular fibrillation with periods of normal sinus rhythm that returns to ventricular fibrillation shortly after conversion or during resuscitation) may be "practically" the same, but others have suggested refractory ventricular fibrillation, and recurrent ventricular fibrillation may, in fact, be two separate entities requiring two separate approaches [1]. Until there are clear treatment differences between recurrent and refractory ventricular fibrillation, DSD can and should be, considered for patients when traditional ACLS algorithms do not result in resolution or improvement of a patient's cardiac dysfunction.

As detailed previously, DSD is not a proven treatment method at this time, but there have been several documented successes across multiple facilities and treatment locations (pre-hospital and within the ED) with its use. Formal studies to evaluate the efficacy of DSD should be investigated to provide further support to patients with refractory ventricular fibrillation not improved by traditional ACLS algorithms. Until formal studies can be completed we recommend the following when a patient presents with refractory ventricular fibrillation:

Dual sequential defibrillation can be attempted after three unsuccessful defibrillation attempts with increasing joules; attempt 1, 150 J, attempts 2 and 3, 200 J (or higher for the third attempt depending on the maximum energy setting of the defibrillator unit).

The orientation of pads should be placed in the conventional orientation (right upper chest and left lateral lower chest) with additional pads placed in the anterior-posterior orientation as detailed in Figure 5.

The same user should activate both electrical shocks at the same time (as close as possible).

Traditional ACLS algorithms should always be followed before attempting DSD.

## Conclusions

Refractory ventricular fibrillation is a rare but deadly condition without clear recommendations once ACLS algorithms have been exhausted. DSD is an option that can be utilized with documented success in a variety of clinical settings and locations. We recommend the use of DSD after three unsuccessful defibrillation attempts and failure of traditional ACLS algorithms to provide resolution of refractory ventricular fibrillation.
